# Supplementary material for: The Effects of Different Processing Methods on the Levels of Biogenic Amines in Zijuan Tea
Source: Foods. 2022 Apr 27;11(9):1260. doi: 10.3390/foods11091260 (PMC9103763; doi:10.3390/foods11091260)
Supplement: Supplementary file 1 [file foods-11-01260-s001.zip › foods-1688052-supplementary.pdf]

Table S1. The contents of biogenic amines (μg/g) in the Zijuan tea.

Samples	Original leaves	Green tea	White tea	Black tea	Dark tea
Methylamine	58.68±0.99	19.04±0.43	21.06±0.38	10.21±0.50	23.60±0.72
Ethylamine	68.23±1.38	516.07±20.48	550.18±8.44	757.97±19.84	19.63±1.67
Pyrrolidine	—	47.03±0.73	29.63±0.95	—	—
Butylamine	78.87±4.20	87.18±1.84	80.78±1.19	—	44.37±0.64
2-Phenethylamine	8.31±1.32	25.85±1.63	31.86±1.48	14.03±1.58	26.31±2.09
Putrescine	—	36.14±2.20	—	—	—
1,7-Diaminoheptane	37.23±0.66	36.48±0.74	44.64±0.87	35.45±2.76	30.38±0.55
Histamine	27.72±1.56	73.22±3.77	50.30±2.02	24.38±1.01	16.90±0.87
Tyramine	26.22±0.77	27.13±1.16	14.46±1.55	8.78±0.84	—
